# AutoDock-SS: AutoDock for Multiconformational Ligand-Based Virtual Screening

**DOI:** 10.1021/acs.jcim.4c00136

**Published:** 2024-04-16

**Authors:** Boyang Ni, Haoying Wang, Huda Kadhim Salem Khalaf, Vincent Blay, Douglas R. Houston

**Affiliations:** † Institute for Quantitative Biology, Biochemistry and Biotechnology, University of Edinburgh, Edinburgh EH9 3BF, U.K.; ‡ Department of Microbiology and Environmental Toxicology, University of California at Santa Cruz, Santa Cruz, California 95064, United States

## Abstract

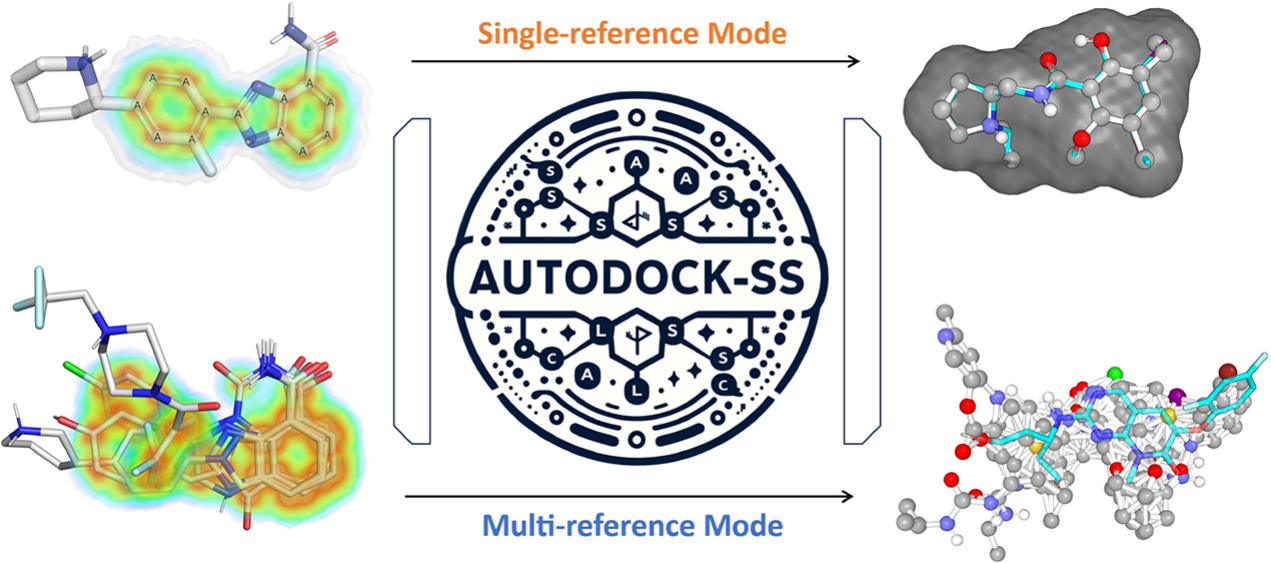

Ligand-based virtual screening (LBVS) can be pivotal for identifying potential drug leads, especially when the target protein’s structure is unknown. However, current LBVS methods are limited in their ability to consider the ligand conformational flexibility. This study presents AutoDock-SS (Similarity Searching), which adapts protein–ligand docking for use in LBVS. AutoDock-SS integrates novel ligand-based grid maps and AutoDock-GPU into a novel three-dimensional LBVS workflow. Unlike other approaches based on pregenerated conformer libraries, AutoDock-SS’s built-in conformational search optimizes conformations dynamically based on the reference ligand, thus providing a more accurate representation of relevant ligand conformations. AutoDock-SS supports two modes: single and multiple ligand queries, allowing for the seamless consideration of multiple reference ligands. When tested on the Directory of Useful Decoys—Enhanced (DUD-E) data set, AutoDock-SS surpassed alternative 3D LBVS methods, achieving a mean AUROC of 0.775 and an EF_1%_ of 25.72 in single-reference mode. The multireference mode, evaluated on the augmented DUD-E^+^ data set, demonstrated superior accuracy with a mean AUROC of 0.843 and an EF_1%_ of 34.59. This enhanced performance underscores AutoDock-SS’s ability to treat compounds as conformationally flexible while considering the ligand’s shape, pharmacophore, and electrostatic potential, expanding the potential of LBVS methods.

## 1 Introduction

Virtual screening (VS) is growing in usefulness as a technique in the early stages of drug discovery, enabling computational prefiltering of large compound libraries to identify potential bioactive compounds for experimental testing.^1^ Although VS is efficient, there is an increasing need to enhance the accuracy of VS methods to reduce false positives and improve true positive rates.^1,2^

VS methods range from structure-based virtual screening (SBVS), which leverage the known three-dimensional structure of the target as in molecular docking,^3^ to ligand-based virtual screening (LBVS), which is employed when the target structure is unknown but initial binding compounds have already been identified. LBVS methods can be mainly classified into fingerprint-based similarity,^4^ machine learning-based methods,^5^ quantitative structure–activity relationship (QSAR),^6^ shape similarity methods,^7^ or ligand-based pharmacophore.^8,9^ The rationale behind LBVS is the Similar Property Principle, which indicates that structurally similar molecules often have similar properties.^10^ 3D LBVS emphasizes three-dimensional similarity to account for the ligand conformations critical for binding to the target’s pocket to initiate key interactions. Established 3D LBVS methods include OptiPharm,^11^ ROCS,^12^ SHAFTS,^13^ and Shape-IT.^14^ Ultrafast Shape Recognition (USR)^15^ is another method that utilizes vectorized descriptors to define molecular 3D shapes. Some 3D LBVS approaches like USRCAT^16^ and eSim^17^ further improve accuracy by combining shape similarity with molecular chemical properties such as pharmacophores or electrostatic potential. For 3D LBVS methods, pregeneration of conformers is essential. Ligands can exist in multiple conformations, and it is significant to explore these different conformations to understand how they might interact with potential drug targets. Pregeneration of conformers helps in accounting for this conformational flexibility during the virtual screening process and increase the likelihood of identifying bioactive structures.^18^ Typical conformer generation does not consider the structure of a reference molecule but rather generates conformers based on the lowest energy or statistically more probable conformations for each compound. If the generated conformers could be optimized based on the structure of a reference molecule, the likelihood of identifying bioactive conformations could be enhanced.

To address these limitations, we developed an innovative LBVS workflow incorporating the molecular docking tool, AutoDock.^19^ AutoDock excels in studying protein–ligand interactions through its precalculated volumetric grid map, streamlining interaction energy calculations. While advantageous, AutoDock’s prolonged execution time in AutoDock4 constrains large-scale use. This was mitigated in AutoDock-GPU,^20^ an OpenCL implementation of AutoDock4 leveraging GPU architecture for accelerated processing. Building on these advances, we present AutoDock-Similarity Searching (AutoDock-SS), a novel 3D LBVS workflow integrating AutoDock-GPU. AutoDock-SS generates ligand-based grid maps from reference ligands rather than proteins with two modes available for single or multiple references. With its integrated conformational sampling, AutoDock-SS emerges as the first LBVS method to intrinsically treat compounds as conformationally flexible without requiring prior conformer generation.

## 2 Methodology

### 2.1 Hardware and Software

The workflow was developed on a server running Ubuntu 18.04.6 LTS with a GNU/Linux 5.4.0–65-generic x86_64 kernel. The server was equipped with four AMD EPYC 7642 central processing units (CPUs) providing 96 cores with two available threads per core, four NVIDIA GeForce RTX 3090 graphics processing units (GPUs), and 320 GB of random-access memory (RAM).

Python 3.9.12 was used with package management via Conda 4.13.0. Key Python packages included RDKit 2022.03.2,^21^ NumPy 1.22.3,^22^ SciPy 1.0.2, Matplotlib 3.5.1, and AutoDockTools_py3 (available at https://github.com/Valdes-Tresanco-MS/AutoDockTools_py3). Additionally, OpenBabel 3.1.0^23^ and the Molecular Graphics Laboratory Tools 1.5.7 (MGLTools) graphical user interface were utilized.

The workflow centered on AutoDock-GPU 1.5.3 compiled with CUDA 11.5 acceleration. The workgroup size was 128 with multithreading enabled. Ligand-protein docking used 100 Lamarckian genetic algorithm (LGA) runs per compound, outputting results as docking log files. Remaining parameters were default values.

Other software packages used were PyMOL 2.5.7 for analysis, visualization, and figure generation and VEGA ZZ for molecular volume and surface area calculations.

### 2.2 Preparation of Reference Ligands and VS Library

#### 2.2.1 Single Reference Mode

The input reference ligand should be provided in its bioactive conformation for optimal LBVS. The protonation state of the reference ligand is corrected at pH = 7.4 by using OpenBabel. Nonpolar hydrogen atoms are removed and Gasteiger partial charges added using MGLTools. The processed reference ligand is output in the PDBQT format with all torsional freedom removed for the AutoDock-SS pipeline.

#### 2.2.2 Multi Reference Mode

Multiple aligned reference ligands can be obtained by extracting ligands from superimposed protein–ligand complexes. Alternatively, known reference ligands could be first aligned with molecular alignment algorithms such as BCL::MolAlign.^24^ Alternatively, aligned references can be obtained by extracting ligands from superimposed protein–ligand complexes. In this study, we used the former method. Nonpolar hydrogens are removed, partial charges added, and output as PDBQTs. AutoDock-SS superimposes the PDBQTs into a single reference complex, which serves as the new multifeature reference ligand. The concentration of multiple reference atoms of the same type in certain positions in space may indicate the pharmacophore. Grid points near these pharmacophores have more negative pseudopotential values (see below). As a result, query molecules whose atom types and locations match those of the pharmacophore naturally receive a boost in their AutoDock-SS scores.

#### 2.2.3 VS Library Preparation

Compounds in the VS library are combined into a single structure-data file (SDF), which is the valid input format for AutoDock-SS.

### 2.3 Generation of Ligand-Based Grid Maps

Ligand-based grid maps are created for each atom type (affinity maps) along with an electrostatic potential map.^19^ Determining the grid box size and center point is essential for building the maps. The 3D coordinates of the reference ligand atoms are obtained using RDKit to calculate the centroid to be used as the box center. The grid box dimensions account for all of the VS library compounds and the reference ligand. The molecular box size of each compound is its maximum *X*, *Y*, and *Z* extension. The grid box size is defined as the maximum value in each direction, rounded up to the nearest even integer (e.g., (60.5 49.5, 60.0 Å) becomes (62, 50, 60 Å)). Even dimensions are required for AutoDock-GPU compatibility, and rounding up provides an adequate conformational sampling space during screening. With the grid box center and size determined, and a spacing of 0.375 Å between grid points (i.e., AutoDock-GPU default value), the coordinates of all grid points can be calculated.

**Figure 1:**
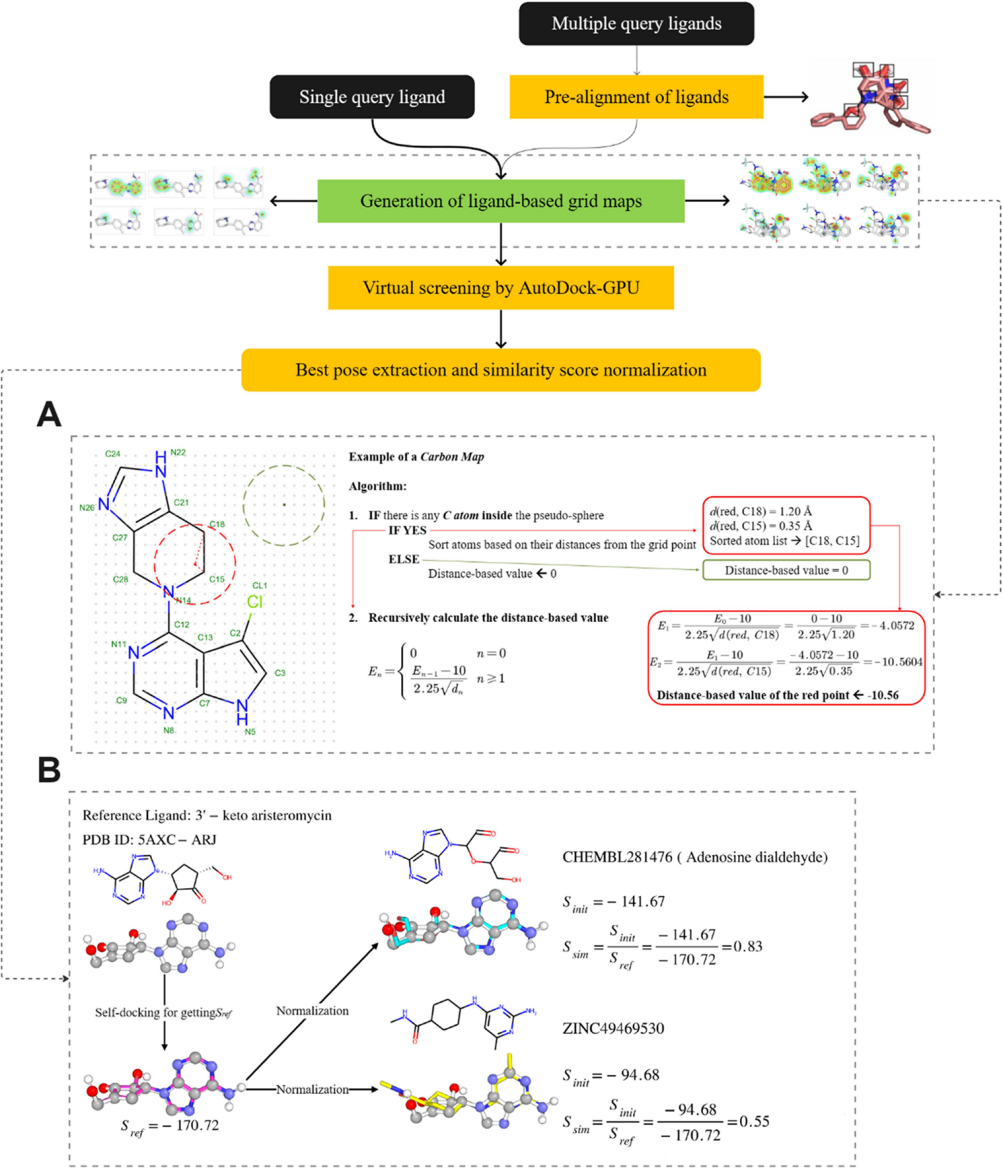
Basic workflow of AutoDock-SS, supporting both a single query ligand and multiple query ligands. Ligand-based grid maps are generated for the single query ligand or the superimposition of multiple query ligands, which are then fed into the AutoDock-GPU for virtual screening. The best pose is extracted and the similarity score is calculated. (A) 2D representation of grid points from a carbon map and corresponding pseudospheres that, along with eq 1, are used to calculate the pseudopotential value of this point for a specific atom type. A list of all atom types considered in shown in Table S1. (B) Example demonstrating the use of eq 3 to normalize the *S*_init_ for an active compound and a decoy. The ball-and-stick structure represents the reference ligand 5AXC-ARJ, and the structure in magenta is the reference ligand itself. The left bottom corner shows the reference ligand self-docking into its own maps (not shown) to obtain the *S*_ref_. The two structures on the right exemplify the best conformations extracted, their corresponding *S*_init_, and how their *S*_init_ values are normalized to the *S*_sim_.

Similar to traditional grid maps, ligand-based maps store scalar fields, each with a pseudopotential value associated with each grid point and atom type. AutoDock-SS identifies all atom types in the reference ligand and bins the coordinates accordingly. For a specific atom type map, the algorithm loops through all grid points. For each grid point, a pseudosphere of radius 1.54 Å is constructed. If the interested atom type is absent in the sphere, the pseudopotential value is 0. Otherwise, atoms in the sphere are distance-sorted, and a pseudopotential is calculated by the equation below (eq 1):
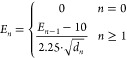
1Here, *n* is the atom index from the sorted list, *E*_*n*_ is the updated pseudopotential contribution from the *n*^th^ atom, and *d*_*n*_ is the distance between the sphere center and *n*^th^ atom. The algorithm iterates through the list, returning the final grid point’s pseudopotential value for a specific atom type (see Figure 1A for an example).

**Figure 2:**
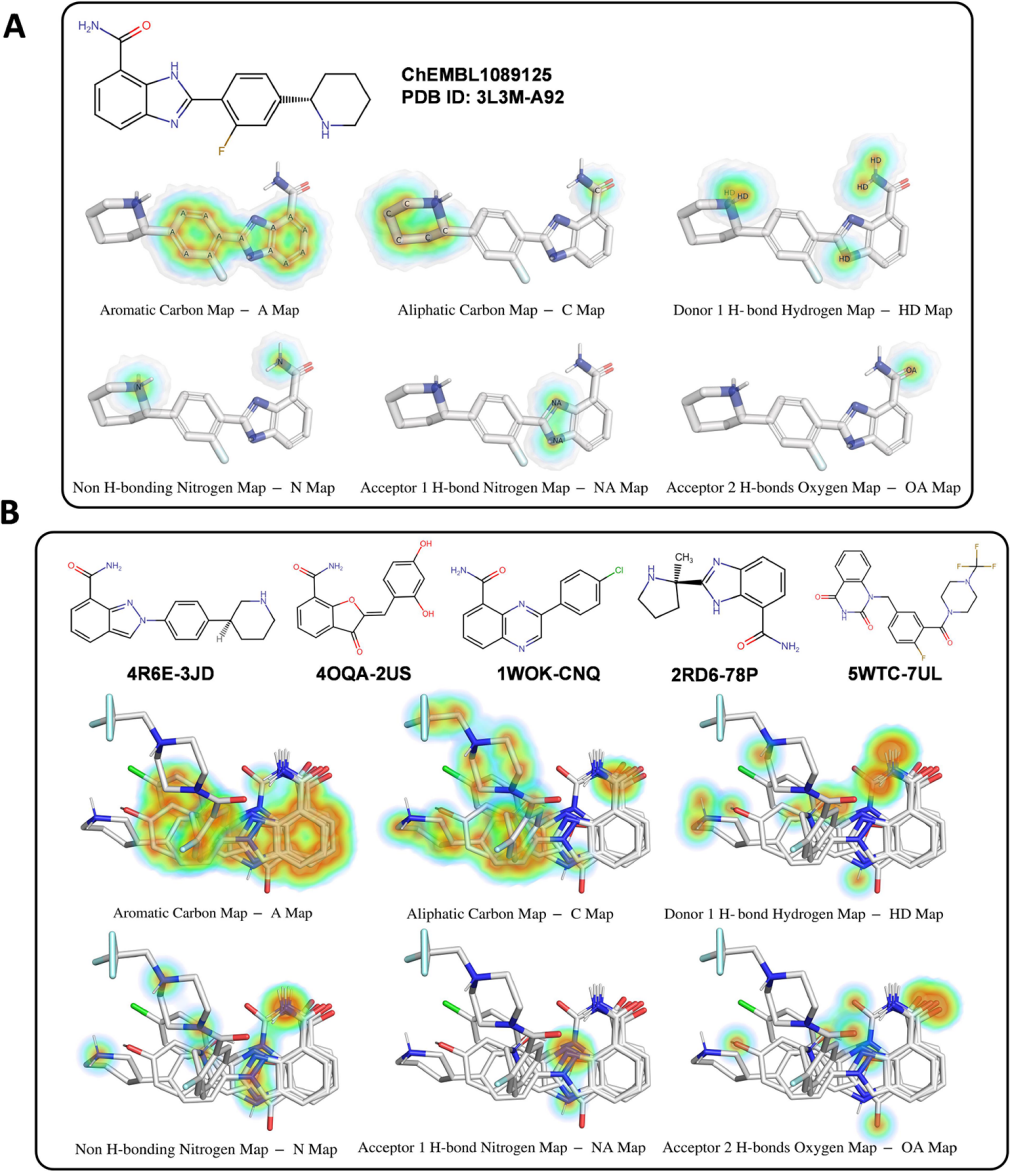
(A) Reference ligand-based grid maps (affinity maps) generated for different atom types of 3L3M-A92. The affinity map for the fluorine atom is not presented here as it is in the same single-atom format as the OA map. (B) Grid maps based on the superposition of five aligned ligands. For each affinity map, the corresponding atom type is labeled on each atom. The affinity maps have a gradient based on the distance to the atom and the number of atoms in the pseudosphere. The deeper the color, the more favored the region for that atom type.

A grid point in a specific atom map will have a more negative value, indicating a preferred position for that atom type during screening, if (1) it is closer to that atom type or (2) there are more of that atom type in its pseudosphere. This evidence the potential of multireference maps to extract pharmacophoric features from multiple ligands. Figure 2 shows the 3D representation of maps generated for the reference ligand atom types, with deeper colors as more preferred regions.

While informative maps are built only for the reference ligand atom types, general maps are required for all possible atom types in AutoDock-GPU. For example, if a VS library compound contains an Cl atom but the reference does not, screening will fail with an error without a general Cl map. Table S1 lists all required atom types and symbols. General maps have identical grid points as informative maps, but all points are set to zero to avoid affecting results.

**Figure 3:**
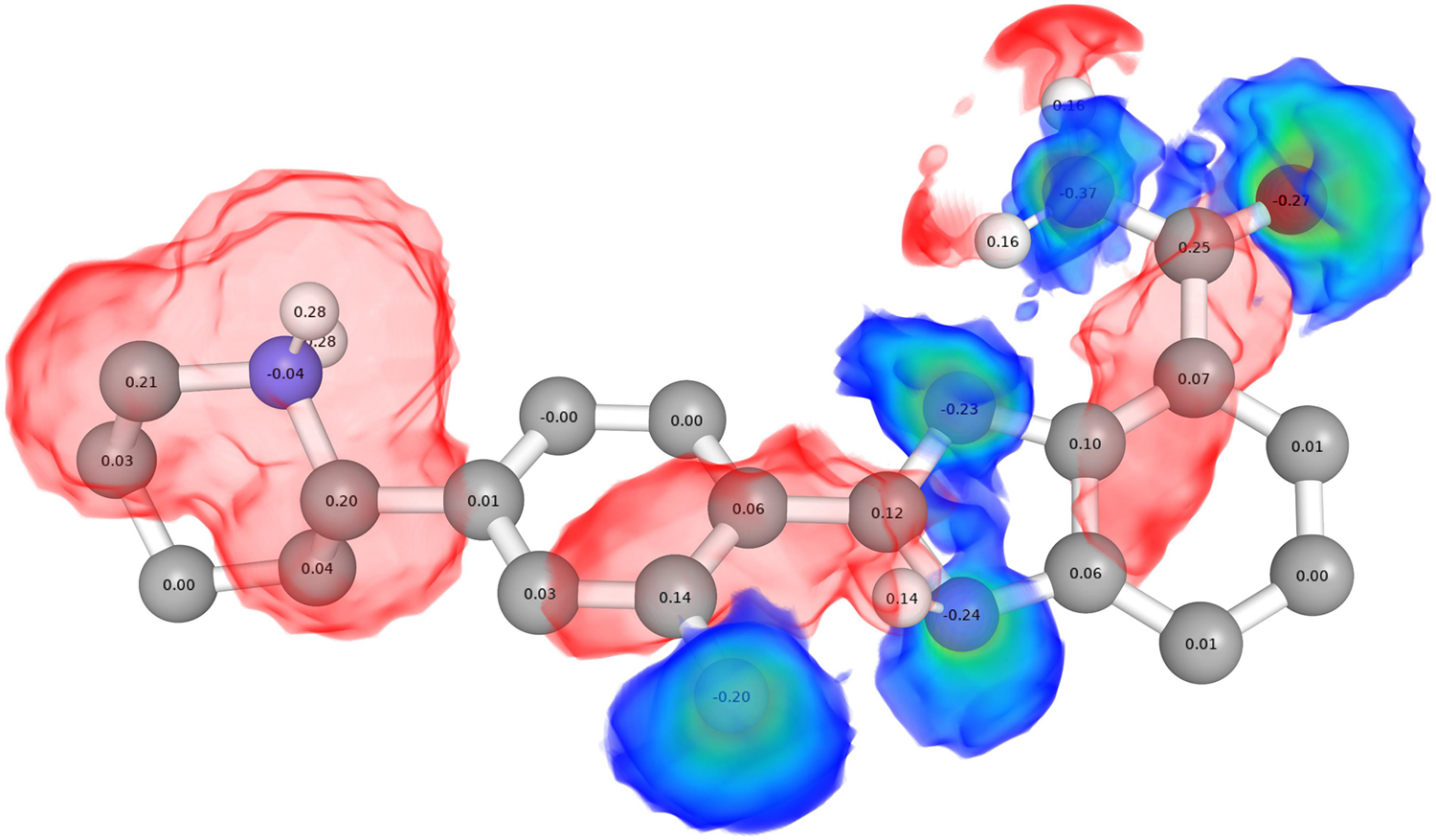
Electrostatic potential map of the ligand ChEMBL1089125, with atomic partial charges labeled on all atoms. The blue regions with gradient represent the preferred areas for negatively charged atoms, and different shades stand for different levels of preference (the deeper, the more preferred). The red regions indicate the preferred areas for positively charged atoms; a gradient for these areas is not presented here.

The electrostatic potential map uses the pseudosphere concept without considering specific atoms. When any atom is in a grid point’s pseudosphere, the electrostatic potential is calculated as (eq 2):
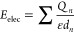
2Here, *n* is the number of atoms in the pseudosphere, *Q*_*n*_ is the partial charge of atom *n*, *d*_*n*_ is the distance between the grid point and atom *n*, and ε is the dielectric constant, using AutoDock4’s default value −0.1465. Figure 3 shows the electrostatic potential map for the reference ligand (the same as the one in Figure 2A), with partial charges labeled. Blue regions prefer negatively charged atoms, while red regions favor positively charged ones.

### 2.4 AutoDock-GPU Virtual Screening and Best Pose Extraction

In standard protein–ligand docking, AutoDock-GPU uses an internal semiempirical force field to estimate the binding energy between the ligand and protein. This accounts for six pairwise energy terms and an estimation of the conformational entropy lost upon binding. The pairwise evaluations include dispersion/repulsion, hydrogen bonding, electrostatics, and desolvation.^25,26^ Notably, the desolvation map is excluded in this study, as it is difficult to generate from only the reference ligand.

In AutoDock-SS, the reference ligand plays the protein role in the docking process. Since 100 Lamarckian genetic algorithm runs are used, 100 optimal conformations fitting the grid maps are output for each screening compound, with the corresponding ADSS scores indicating map fit. From the resulting conformations, the pose with the lowest score (*S*_init_) is returned. More negative *S*_init_ values indicate higher similarity to the reference ligand(s).

### 2.5 Similarity Score Calculation

The initial score (*S*_init_) can vary, depending on the reference ligand. To normalize this score, the reference ligand is self-docked into its maps to obtain a reference score (*S*_ref_) for normalization. The ADSS similarity score (*S*_sim_) between a VS compound and reference is (eq 3):
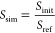
3

Normalization assumes higher *S*_sim_ indicates greater similarity to the reference. Figure 1B illustrates the *S*_init_ normalization. The ball-and-stick visualization corresponds to the reference molecule in its bioactive conformation. The *S*_ref_ was calculated by docking the reference ligand (magenta) into its own maps and then used to normalize the *S*_init_ of the VS library compounds (cyan and yellow). Self-docking (magenta) nearly recovered its active conformation, suggesting that *S*_ref_ is theoretically the ceiling of *S*_init_. Therefore, the range of the similarity score is [0, 1]. The larger *S*_sim_ of the cyan compound indicated a greater similarity, visually confirmed by the alignments.

While *S*_sim_ represents ligand similarity, it essentially reflects the map fit. A compound can have multiple *S*_sim_ values for different conformations, but the maximum *S*_sim_ (minimum similarity value) pose is ultimately extracted. For the multireference mode, we define *S*_sim_ as the quotient of *S*_init_ divided by the sum of *S*_ref_ values for all the reference ligands.

### 2.6 Performance Evaluation

#### 2.6.1 DUD-E Database

DUD-E is a standard data set for evaluating structure-based and 3D ligand-based methods, with actives and decoys designed to have low 2D similarity.^27^ It contains 102 protein targets, 22,886 actives, and ∼50 decoys per active. The data set provides protein structures (PDB), native ligand structures (SYBYL mol2), and active and decoy sets (SDF). Native ligands were used as references and prepared as described. AutoDock-SS was evaluated on the full DUD-E data set. Notably, our Supporting Information includes an analytical investigation on the relationship between the degrees of freedom (DOF) of library compounds and the performance of AutoDock-SS, where the number of rotatable bonds (NRotB) was used as a proxy for DOF.

#### 2.6.2 DUD-E^+^ Database

DUD-E^+^ was designed to test the effect of target ligand choice on LBVS methods.^17^ It comprises 92 of the 102 protein targets in DUD-E, each of which contains five alternative protein structures that are mutually aligned along with their corresponding ligands. Formats are identical with those of DUD-E, with five additional aligned native ligand structures provided. These were directly utilized as references.

#### 2.6.3 Evaluation Metrics

The area under the receiver operating characteristic curve (AUROC) measures the ability to distinguish actives from decoys. AUROC of 1.0 indicates perfect classification, while ∼0.5 signifies random performance. Higher AUROC values indicate greater predictive accuracy.

Enrichment factor (EF) evaluates active enrichment among top-scoring hits versus overall concentration, which is calculated by (eq 4):
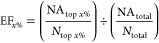
4where NA_top *x*%_, *N*_top *x*%_, N*A*_total_, and *N*_total_ are the number of actives in the top *x*_%_ of the screened library, the total number of compounds in the top *x*_%_ of the screened library, the total number of actives in the entire library, and the total number of compounds in the entire library, respectively. Generally, because only the top hits of virtual screening are of interest, this study focused on evaluating the early enrichment of AutoDock-SS. Therefore, the EF at 1% (EF_1%_) was used as the primary measure.

## 3 Results and Discussion

Two performance analyses were conducted to characterize the prediction accuracy of AutoDock-SS.

1. Single-reference mode: the first analysis compared the AUROC values and computational speed performance between AutoDock-SS and 9 different LBVS methods. Importantly, direct comparisons with structure-based/docking approaches were conducted. We have now included performance data for AutoDock-GPU, derived from experiments conducted on the same DUD-E entries, spacing values and numbers of grid points used for AutoDock-SS. Additionally, performance data from the literature concerning AutoDock-Vina and AutoDock-Vina AD4 map was incorporated. Notably, all performance data for non-AutoDock-SS and non-AutoDock-GPU methods were collected from the cited literature.
2. Multireference mode: the second analysis assessed the performance of AutoDock-SS, using a superimposed reference complex of five mutually aligned ligands as the query against the DUD-E^+^ data set. This analysis compared the AUROC and enrichment factor at 1% (EF_1%_) values between the single-reference and multireference modes.

Together, the analysis provided a robust assessment of AutoDock-SS accuracy and highlighted strengths and potential improvements.

### 3.1 AutoDock-SS Single-Reference Mode Performance on the DUD-E Data Set

**Figure 4:**
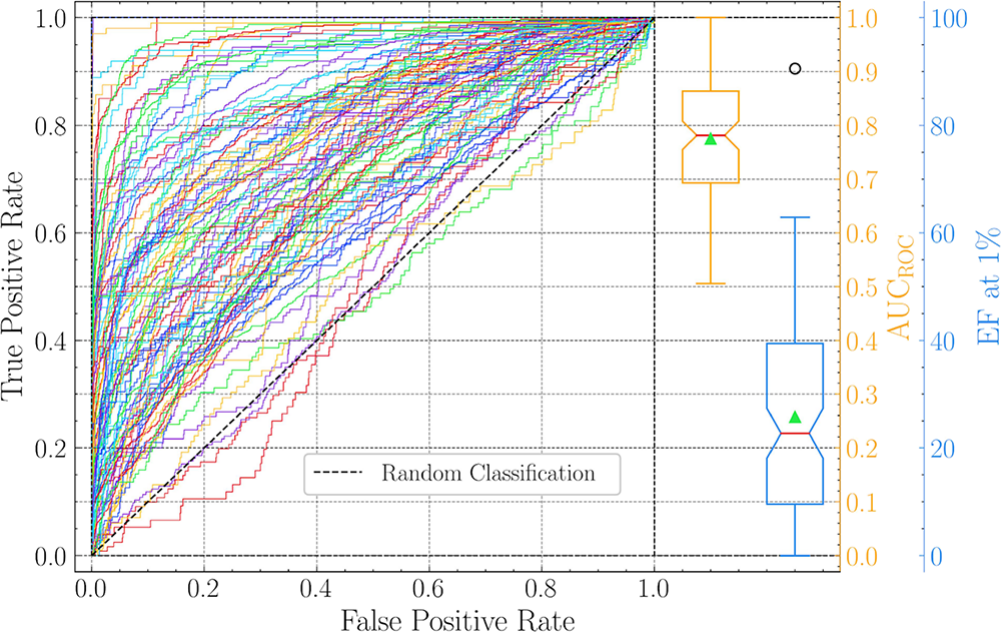
Cluster of 102 ROCs from the evaluation of AutoDock-SS single reference mode performance in screening all DUD-E targets. The orange boxplot on the right represents the distribution of AUROC values, and the blue one shows the distribution of EF_1%_ values. The red line in the boxplot indicates the median value, and the green triangle represents the mean value.

Table S2 provides the detailed AutoDock-SS single reference mode results for the full DUD-E set. The mean AUROC of single-reference mode was 0.775 ± 0.123, and the mean EF_1%_ was 25.72 ± 18.66 (i.e., 25.72 times as many actives were detected than were by a random pick in the first 1% of the ranked data set). The cluster of all ROCs for all 102 targets and boxplots indicating the distribution of AUROC and EF_1%_ values are presented in Figure 4.

Table 1 compares AutoDock-SS single reference performance to 9 other 3D LBVS methods, along with AutoDock-GPU, AutoDock-Vina, and AutoDock-Vina-AD4, using literature data for eSim, ROCS, USR, WEGA, OptiPharm, and AutoDock-Vina variants. AutoDock-SS single-reference mode achieved the highest mean AUROC on full DUD-E screening, outperforming both state-of-the-art ligand- and structure-based techniques. It had the most cases above the AUROC of 0.6, the fewest below the AUROC of 0.5, the most stable performance across all 102 targets, and exceptional early enrichment ability. This demonstrates AutoDock-SS’s strength in distinguishing actives from computationally designed decoys and meaningfully enriching hits for real lead discovery. In summary, benchmarking shows that the AutoDock-SS single reference mode surpasses leading 3D LBVS methods, combining high accuracy in separating challenging actives and decoys with superior early enrichment.

**Table 1:** Summary of Performance Values of Ten LBVS Methods (All Variants Included) and AutoDock Variants on the Full DUD-E Dataset of 102 Targets^a^

method	mean AUROC	AUROC < 0.50	AUROC ≥ 0.60	AUROC ≥ 0.70	AUROC ≥ 0.80	AUROC ≥ 0.90	AUROC ≥ 0.95	speed (mols/s)
AutoDock-SS single-reference mode	0.775	0	95	75	44	18	9	5.9
eSim -pscreen	0.755	5	81	69	43	17	8	12.3
eSim -pfast	0.736	9	82	62	34	14	5	61.2
eSim -pfastf	0.663	5	79	53	26	6	3	274.9
ROCS	0.596	18	66	44	21	9	3	50
ROCS (shape)	0.570	31	54	25	12	1	0	50
WEGA	0.560	31	44	19	0	0	0	26.7
OptiPharm (robust)	0.560	32	41	15	0	0	0	12
USR	0.554	35	43	20	1	0	0	6000
USR (shape)	0.520	43	28	13	0	0	0	6000
AutoDock-GPU	0.681	7	80	44	14	4	1	4.5
AutoDock-Vina	0.720	3	88	66	30	5	1	N/A
AutoDock-Vina-AD4	0.700	5	81	56	23	5	2	N/A

a Note: performance data for eSim were taken from,^17^ data for WEGA and OptiPharm were taken from,^11^ data for ROCS and USR were taken from,^28^ and data for AutoDock-Vina and AutoDock-Vina AD4 map were taken from ref (29). Notably, the computational speed is directly related to the hardware configuration employed. The speeds listed in the table are based on various hardware setups. Please refer to the literature cited for detailed configurations. Brief description of mentioned LBVS methods: (1) eSim combines electrostatic field comparison with molecular surface shape and hydrogen bonding directionality. It calculates similarity using feature values at observer points around a query ligand focusing on steric distances, Coulombic energies and hydrogen bond characteristics. (2) ROCS leverages Gaussian functions to assess shape similarity between molecules, which builds on the Gaussian overlay algorithm, identifying the greatest volume overlap between structures. (3) WEGA introduces weighted atomic Gaussian functions to correct the overestimation of overlap volumes found in previous methods. (4) OptiPharm utilizes an evolutionary algorithm for global optimization, focusing on maximizing the Gaussian volumetric overlap between molecules. (5) USR compresses 3D molecular shape into a set of numeric molecular descriptors.

AutoDock-SS’s superior performance can be attributed to two key factors:

First, the adapted reference ligand-based grid maps capture shape and pharmacophore features and describe the shape differently than volume- or surface-based methods. The grid points trace relative atom positions and create preferred regions based on distance. This considers spatial arrangements and the underlying steric and pharmacophoric properties. Together with the electrostatic potential map, three properties were factored into the similarity calculation, while most other 3D shape-based methods consider one additional chemical property of the shape to improve their performance.

**Figure 5:**
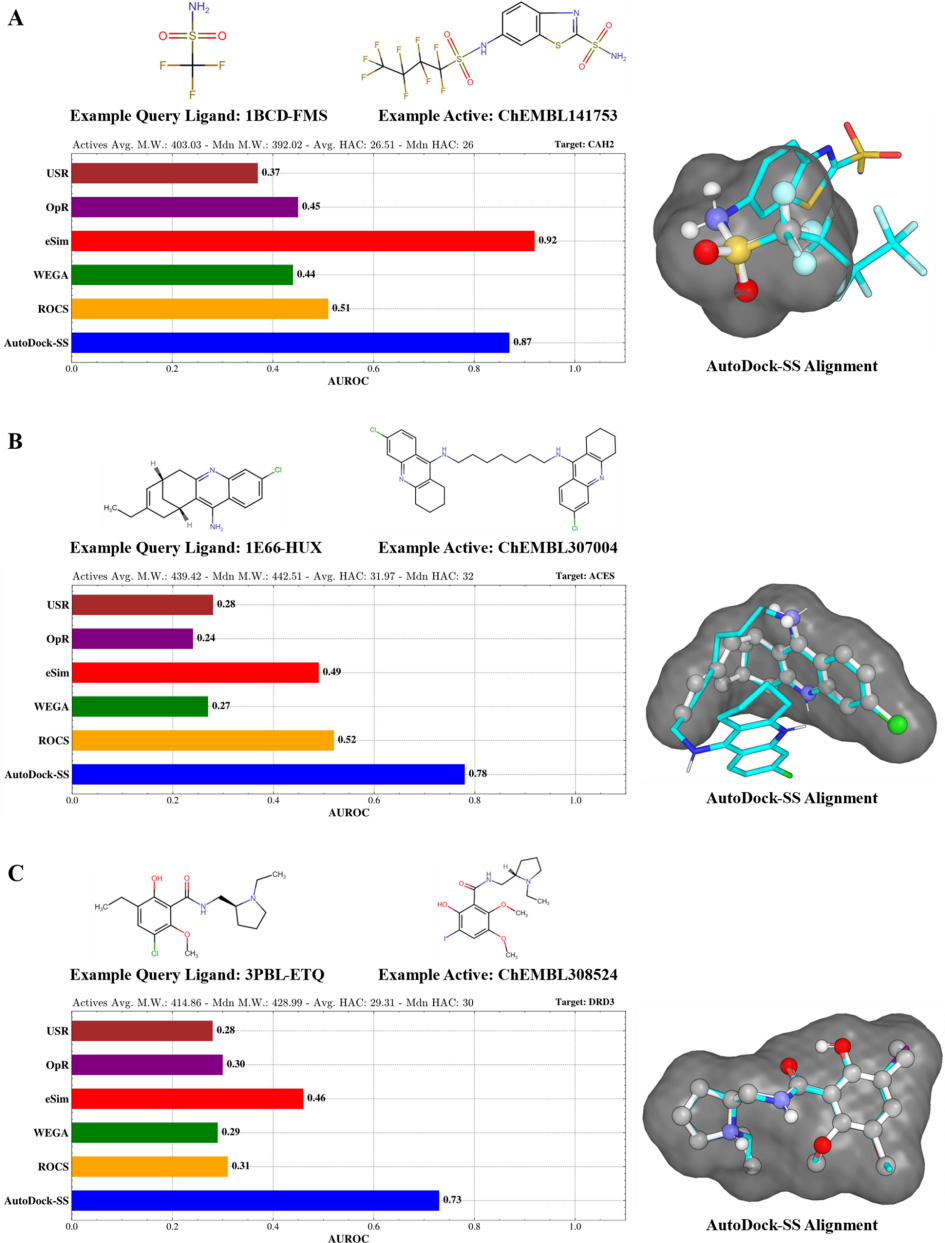
Examples of AutoDock-SS single-reference mode performance compared with alternative methods. Ball-and-stick structures represent the reference ligand, and cyan structures represent example actives. The average and median molecular weights and heavy atom counts of the actives library are displayed. The gray surface represents the area of the ensemble of grid maps (i.e., areas where grid maps have negative pseudopotential values).

Second, removing positive grid point pseudopotentials enables AutoDock-SS to handle significant molecular size differences between the VS library compounds and references, especially when the references are smaller. The traditional grid maps generated from proteins for molecular docking contain both negative and positive values, which represent preferred and nonpreferred regions, respectively. However, some large library compounds possessing active pharmacophores or essential substructures are inadvertently penalized due to the outlier structures. In the DUD-E CAH2 and ACES examples (Figure 5A,B), CAH2’s reference ligand is a third the size of actives, yet AutoDock-SS achieved excellent retrieval aligning on the sulfonamide substructure. This ability is also demonstrated in the acetylcholinesterase (ACES) example, where library actives are approximately 1.5-fold larger in volume than the reference ligand.

Although the size discrepancy probably contributed to the noticeable performance disparities, it did not account for all performance variations observed. Despite numerous targets for which the volume of the reference ligand and the average volume of the actives were close, the AutoDock-SS single-reference mode still displayed similar performance advantages. The DRD3 case (Figure 5C) is an example of AutoDock-SS performing significantly better even when the query ligand and actives have similar sizes.

Regarding computational speed, AutoDock-SS can be slower than other 3D LBVS methods, even with GPU acceleration and multithreading because docking procedures and modeling flexibility require additional time for conformational sampling. Notably, screening rigid DUD-E was 2.5× faster versus the flexible one. With four RTX3090 GPUs, AutoDock-SS achieved a speed comparable to eSim-pscreen. Speed is also influenced by average query and library compound sizes and by torsional degrees of freedom, which determine the grid dimensions and conformational space.

Despite a slower speed, AutoDock-SS is still valuable and can be accelerated to match other methods given sufficient GPU resources. Critically, it provides superior accuracy and early enrichment, which is essential for drug discovery. The trade-off of reduced speed for higher performance merits exploration where resources permit.

In summary, conformational sampling leads to slower speed, but this can be easily overcome with additional GPU resources. In return, AutoDock-SS offers beyond-state-of-the-art accuracy and enrichment in LBVS.

### 3.2 AutoDock-SS Multireference Mode Performance on DUD-E^+^ Data set

AutoDock-SS multireference mode screened all 92 DUD-E^+^ targets (Table S2). The mean AUROC value was 0.843 ± 0.111, and the mean EF_1%_ was 34.59 ± 19.55, which increased by approximately 9 and 35%, respectively, compared to the single-reference mode. Table S2 highlights cases with AUROC differences >0.1. The multireference mode demonstrated significantly better performance than the single reference mode in 27 cases, while the opposite occurred in only three cases. A paired *t* test gave a two-tailed *P*-value <1 × 10^–5^ between the two modes, indicating that the multireference mode is statistically superior. With four RTX 3090 GPUs and 192 CPU threads on prealigned ligands, multireference throughput was 5.8 compounds/second, slightly slower than single reference.

**Figure 6:**
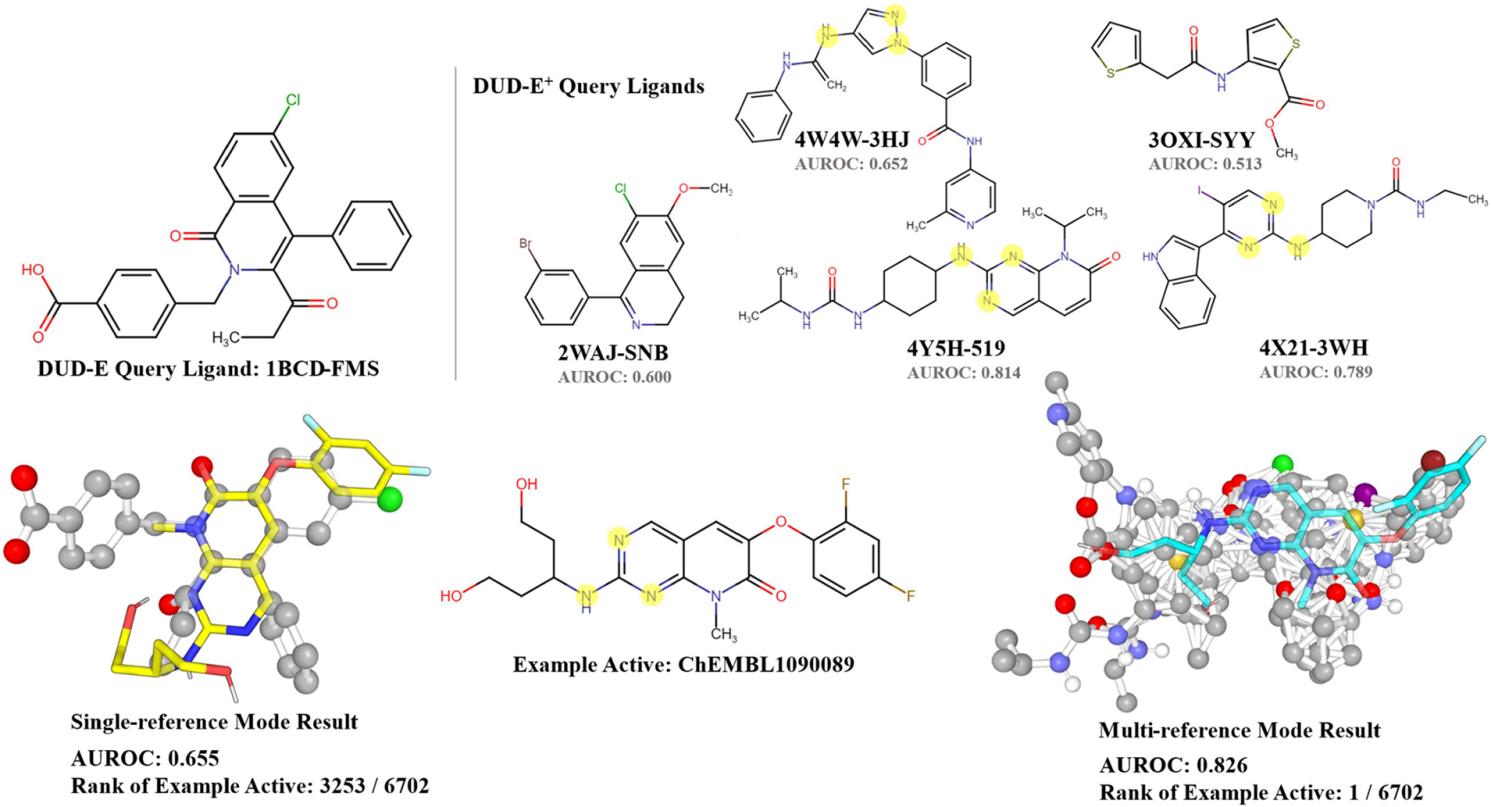
Example of different predictions made by the multireference mode and the single-reference mode based on the superimposition of five reference ligands and a single reference ligand, respectively. The yellow structure shows the best conformation predicted by the single-reference mode, and the cyan structure was predicted based on the mutual alignment of the five ligands. The ball-and-stick structures represent the reference ligands and the superimposition of five query ligands. The additional features presented in the multireference mode are highlighted. The AUROC value for each DUD-E^+^ ligand is indicative of the performance of the multireference complexed by that ligand and the default DUD-E ligand.

The enhanced performance of the multireference mode can be attributed to two main factors. First, using multiple reference ligands allows for a more comprehensive representation of possible atomic positions. While the single-reference mode relies on a single reference ligand, it cannot account for all atomic positions of different actives under real protein–ligand binding conditions. Second, the overlapping regions representing potential pharmacophores have amplified pseudopotential values. As in Figure 1, clustered atom types likely indicate an essential pharmacophore, with increased negative energies proportional to atom numbers in those grid points. Figure 6 shows the MK10 case, with AUROC increasing from 0.655 (single-reference mode) to 0.826 (multireference mode). Following this, Figure 6 also elucidates the impact of incorporating one additional reference ligand on the accuracy. The disparate AUROC values −0.600 (2WAJ-SNB), 0.513 (3OXI-SYY), 0.652 (4W4W-3HJ), 0.789 (4X21-3WH), and 0.814 (4Y5H-519)—demonstrates how the unique structural features of each ligand contribute to their efficacy. When a ligand with important pharmacophore, as highlighted in Figure 6 is included, the multireference mode yields a superior performance. The single mode depended on specific atom alignments, while the multireference facilitated better active positioning. However, false positives may increase as decoys can also achieve improved fits. Overall, multireference improved most cases when multiple ligands were available.

In summary, multireference screening provides performance benefits over single reference with minimal speed reduction when multiple actives are available. This showcases the advantage of AutoDock-SS’s ability to leverage multiple ligands.

## 4 Conclusions

In this study, we introduced AutoDock-SS, a novel LBVS approach using adapted ligand grid maps and AutoDock-GPU, supporting single or multiple prealigned query ligands.

We assessed the performance of the single-reference mode in terms of prediction accuracy, early enrichment of actives, and computational speed on the full DUD-E data set. In comparison to nine state-of-the-art 3D LBVS methods, the single-reference mode achieved the highest prediction accuracy and a promising EF_1%_ value. This superior performance is attributed to the comprehensive information (shape, pharmacophore, and electrostatic potential) contained in the adapted grid maps and the built-in conformational sampling algorithm for a better binding conformation prediction.

Multireference mode evaluation on the full DUD-E^+^ showed significantly improved accuracy and enrichment versus single reference mode. Grid maps based on the multimolecule complex provide additional atomic positions and enhanced pharmacophores. Despite docking and conformational sampling, both modes had nearly identical competitive speeds using GPUs. AutoDock-SS manifests as one of few LBVS methods with GPU acceleration.

In summary, AutoDock-SS is an efficient ligand-based virtual screening approach with built-in conformational sampling for large-scale virtual screening in drug discovery. It demonstrates superior performance with single or multiple query ligands. AutoDock-SS represents a promising new technique for computational drug discovery applications.

## Data Availability

The original data for all screen DUD-E and Augmented DUD-E are presented in Table S2. The source code for AutoDock-SS will be made publicly available and can be accessed at https://github.com/NiBoyang/AutoDock-SS. The main program and its dependencies are developed in Python, and Jupyter Notebook files used for data analysis are included in the directory.

## Supporting Information Available

Atom types required by affinity maps for AutoDock-SS; detailed AUROC and EF_1%_ values of AutoDock-SS single-reference and multireference mode on the full DUD-E data set; detailed AUROC and EF_1%_ values of AutoDock-GPU on the full DUD-E data set; comparative analysis of AUROC and EF_1%_ values between AutoDock-SS single-reference and multireference mode; ROCs cluster of multireference mode on all 92 DUD-E^+^ targets; one additional study on the relationship between degrees of freedom and AutoDock-SS performance (PDF)

## Abbreviations

AUROC: area under the receiver operating characteristic curve
avg. M.W.: average molecular weight
mdn M.W.: median molecular weight
avg. HAC: average heavy atom count
mdn HAC: median heavy atom count
NRotB: number of rotatable bonds
