# Supplementary material for: AutoDock-SS: AutoDock for Multiconformational Ligand-Based Virtual Screening
Source: J Chem Inf Model. 2024 Apr 16;64(9):3779–89. doi: 10.1021/acs.jcim.4c00136 (PMC11094722; doi:10.1021/acs.jcim.4c00136)
Supplement: Supplementary file 1 — ci4c00136_si_001.pdf [file ci4c00136_si_001.pdf]

Title: AutoDock-SS: AutoDock for Multiconformational Ligand-based Virtual Screening

Authors:

1. Boyang Ni, Mr.
   a. Affiliation: Institute for Quantitative Biology, Biochemistry and Biotechnology, University of Edinburgh, EH9 3BF, UK
   b. E-mail: Boyang.Ni@ed.ac.uk
2. Haoying Wang, Mr.
   a. Affiliation: Institute for Quantitative Biology, Biochemistry and Biotechnology, University of Edinburgh, EH9 3BF, UK
   b. E-mail: wanghaoying1995@hotmail.com
3. Huda Kadhim Salem Khalaf, Miss.
   a. Affiliation: Institute for Quantitative Biology, Biochemistry and Biotechnology, University of Edinburgh, EH9 3BF, UK
   b. E-mail: huda_khalaf@hotmail.com
4. Vincent Blay, Ph.D.
   a. Affiliation: Department of Microbiology and Environmental Toxicology, University of California at Santa Cruz, Santa Cruz, CA 95064, USA.
   b. E-mail: vroger@ucsc.edu
5. Douglas R. Houston, Ph.D.
   a. Affiliation: Institute for Quantitative Biology, Biochemistry and Biotechnology, University of Edinburgh, EH9 3BF, UK
   b. E-mail: DouglasR.Houston@ed.ac.uk

Corresponding Author:

Douglas R. Houston, Ph.D.

a. Affiliation: Institute for Quantitative Biology, Biochemistry and Biotechnology, University of Edinburgh, EH9 3BF, UK
b. E-mail: DouglasR.Houston@ed.ac.uk
c. Address: Room 2.12, Waddington 1 Building, King's Buildings, University of Edinburgh, Edinburgh, EH9 3BF, UK
d. Telephone: +44 7986875743

Author Contributions:

B.N. – Methodology development, algorithm design, investigation, validation, writing original draft and revising. H.W., H.K.S.K – Methodology, algorithms design, investigation. V.B. – Methodology, supervision, reviewing manuscript. D.H. – Conceptualization, supervision, reviewing manuscript.

Supporting Information

Table S1 *Atom types required by affinity maps for AutoDock-SS*

Symbol	Atom type	Symbol	Atom type
HD	Donor 1 H-bond Hydrogen	SA	Acceptor 2 H-bonds Sulphur
C	Non-H-bonding Aliphatic Carbon	Cl	Non-H-bonding Chlorine
A	Non-H-bonding Aromatic Carbon	Br	Non-H-bonding Bromine
N	Non-H-bonding Nitrogen	I	Non-H-bonding Iodine
NA	Acceptor 1 H-bond Nitrogen	Ca	Non-H-bonding Calcium
OA	Acceptor 2 H-bonds Oxygen	Mn	Non-H-bonding Manganese
F	Non-H-bonding Fluorine	Fe	Non-H-bonding Iron
Mg	Non-H-bonding Magnesium	Zn	Non-H-bonding Zinc
P	Non-H-bonding Phophorus		

Table S2 *AUROC and $EF_{1\%}$ values of AutoDock-SS single-reference and multi-reference modes*

	**Single-reference**		**Multi-reference**	
**Target**	**AUROC**	**$EF_{1\%}$**	**AUROC**	**$EF_{1\%}$**
aa2ar	0.857	30.13	0.760	21.19
abl1	0.750	12.74	**0.862**	33.79
ace	0.877	43.11	0.908	35.21
aces	0.784	32.50	0.734	37.37
ada	0.705	33.95	**0.851**	50.37
ada17	0.780	30.54	0.868	32.05
adrb1	0.916	44.28	0.929	49.15
adrb2	0.851	40.50	0.904	55.74
akt1	0.765	9.26	0.729	3.43
akt2	0.764	12.08	0.813	24.17
aldr	0.819	38.41	0.760	39.67

Target	Single-reference		Multi-reference	
	AUROC	$EF_{1\%}$	AUROC	$EF_{1\%}$
ampc	0.809	35.16	-	-
andr	0.715	22.03	**0.814**	14.19
aofb	0.508	0.83	**0.766**	12.43
bace1	0.721	7.79	0.816	53.08
braf	0.786	18.53	0.866	20.52
cah2	0.866	1.43	**0.937**	6.93
casp3	0.674	9.13	0.688	30.93
cdk2	0.601	6.77	**0.764**	22.20
comt	0.983	57.32	0.995	82.25
cp2c9	0.518	8.36	-	-
cp3a4	0.557	7.21	-	-
csf1r	0.808	24.87	0.850	29.72
cxcr4	0.787	38.97	-	-
def	0.654	16.82	**0.911**	42.55
dhi1	0.624	10.93	**0.732**	12.14
dpp4	0.808	25.40	0.863	34.63
drd3	0.732	6.69	-	-
dyr	0.680	2.61	**0.971**	70.00
egfr	**0.886**	39.14	0.702	4.80
esr1	0.776	43.02	**0.939**	44.60
esr2	0.812	39.44	**0.924**	44.37
fa10	0.604	12.66	**0.924**	35.38
fa7	0.947	38.68	0.994	57.14
fabp4	0.735	43.40	**0.953**	43.40
fak1	0.973	56.06	0.970	56.06
fgfr1	0.720	1.45	**0.857**	20.32
fkb1a	0.747	20.94	**0.920**	14.57
fnta	0.678	8.80	**0.822**	23.53
fpps	1.000	90.54	1.000	100.07
gcr	0.803	18.73	0.760	19.90
glcm	0.702	1.86	0.748	13.04
gria2	**0.757**	16.55	0.624	8.27
grik1	0.723	14.10	**0.829**	8.27
hdac2	**0.721**	17.91	0.590	14.65
hdac8	0.872	37.93	0.913	32.60
hivint	0.722	7.12	0.662	9.15
hivpr	0.816	36.10	0.812	45.08

	Single-reference		Multi-reference	
**Target**	**AUROC**	**$EF_{1\%}$**	**AUROC**	**$EF_{1\%}$**
hivrt	0.624	11.62	**0.737**	7.74
hmdh	0.873	53.33	0.853	47.70
hs90a	0.716	46.30	0.746	23.15
hxk4	0.939	51.51	0.985	47.12
igf1r	0.820	19.12	-	-
inha	0.671	29.09	0.597	0.00
ital	0.669	18.30	-	-
jak2	0.739	30.19	0.628	9.44
kif11	0.691	23.35	0.775	38.06
kit	0.705	6.07	-	-
kith	0.980	53.68	-	-
kpcb	0.851	46.41	-	-
lck	0.616	10.50	**0.877**	38.65
lkha4	0.947	33.59	0.909	33.00
mapk2	0.923	39.30	0.946	56.42
mcr	0.816	17.33	0.849	10.83
met	0.835	39.41	0.873	67.91
mk01	0.870	60.60	0.872	58.02
mk10	0.655	2.91	**0.826**	18.45
mk14	0.660	5.72	**0.871**	42.45
mmp13	0.936	44.83	0.888	27.49
mp2k1	0.587	2.49	0.797	35.69
nos1	0.506	0.00	0.488	4.06
nram	0.878	3.09	**0.996**	61.82
pa2ga	0.799	14.41	0.796	19.55
parp1	0.841	25.68	0.900	37.53
pde5a	0.679	18.65	**0.803**	29.24
pgh1	0.528	3.61	0.541	7.21
pgh2	0.803	28.07	0.681	7.36
plk1	0.654	10.38	**0.797**	11.33
pnph	0.680	24.34	**0.995**	67.19
ppara	0.970	48.19	0.930	36.07
ppard	0.974	39.63	0.964	46.72
pparg	0.900	46.45	0.901	37.32
prgr	0.625	1.03	**0.884**	29.86
ptn1	0.720	20.08	0.817	11.58
pur2	1.000	58.05	1.000	58.05

Target	Single-reference		Multi-reference	
	AUROC	$EF_{1\%}$	AUROC	$EF_{1\%}$
pygm	0.507	2.64	**0.627**	26.42
pyrd	0.828	46.96	0.847	56.89
reni	0.784	33.78	**0.887**	47.29
rock1	0.699	3.03	0.797	16.15
rxra	0.906	0.00	0.933	27.56
sahh	1.000	58.26	1.000	58.26
src	0.687	10.89	**0.886**	36.30
tgfr1	0.710	3.04	**0.918**	28.13
thb	0.794	42.40	0.809	43.38
thrb	0.797	24.29	0.889	43.12
try1	0.858	13.37	0.906	49.26
tryb1	0.840	14.27	0.847	31.93
tysy	0.702	35.42	**0.876**	55.93
urok	0.938	47.83	0.926	27.33
vgfr2	0.736	17.18	**0.876**	42.71
wee1	0.989	62.87	0.996	62.87
xiap	0.939	46.86	0.937	53.99

Note: If the AUROC value difference between the two modes is greater than 0.100, the higher AUROC value is bold.

Table S3 *AUROC and $EF_{1\%}$ values of AutoDock-GPU*

Target	AUROC	$EF_{1\%}$	Target	AUROC	$EF_{1\%}$
aa2ar	0.577	2.08	hxk4	0.681	4.38
abl1	0.699	26.59	igf1r	0.787	12.29
ace	0.667	3.95	inha	0.556	9.70
aces	0.696	19.24	ital	0.465	1.46
ada	0.329	0.00	jak2	0.528	4.72
ada17	0.589	4.32	kif11	0.747	6.92
adrb1	0.791	16.25	kit	0.677	9.10
adrb2	0.765	18.72	kith	0.660	7.16
akt1	0.750	7.89	kpcb	0.754	20.96
akt2	0.798	25.90	lck	0.711	14.79
aldr	0.567	1.26	lkha4	0.858	30.06
ampc	0.657	2.07	mapk2	0.762	4.03

andr	0.610	3.73	mcr	0.656	9.75
aofb	0.581	1.66	met	0.741	8.49
bace1	0.789	11.68	mk01	0.742	3.87
braf	0.646	1.99	mk10	0.633	2.91
cah2	0.643	6.11	mk14	0.645	5.37
casp3	0.724	18.76	mmp13	0.625	2.63
cdk2	0.688	10.15	mp2k1	0.603	1.66
comt	0.387	0.00	nos1	0.486	0.00
cp2c9	0.597	9.20	nram	0.834	4.12
cp3a4	0.511	1.80	pa2ga	0.716	1.03
csf1r	0.654	1.21	parp1	0.725	9.28
cxcr4	0.582	10.39	pde5a	0.582	2.27
def	0.699	2.97	pgh1	0.565	2.06
dhi1	0.604	3.95	pgh2	0.669	4.83
dpp4	0.854	17.50	plk1	0.594	2.83
drd3	0.649	5.02	pnph	0.757	5.84
dyr	0.664	18.26	ppara	0.672	7.54
egfr	0.610	9.60	ppard	0.603	1.67
esr1	0.781	35.68	pparg	0.761	12.86
esr2	0.768	24.92	prgr	0.649	2.06
fa10	0.866	17.69	ptn1	0.886	18.53
fa7	0.832	10.55	pur2	0.997	53.91
fabp4	0.741	19.53	pygm	0.385	2.64
fak1	0.584	1.02	pyrd	0.702	5.42
fgfr1	0.634	8.64	reni	0.703	27.02
fkb1a	0.741	3.64	rock1	0.761	12.11
fnta	0.673	4.06	rxra	0.832	10.72
fpps	0.431	0.00	sahh	0.739	3.24
gcr	0.615	3.90	src	0.638	5.54
glcm	0.651	1.86	tgfr1	0.712	10.64
gria2	0.668	5.09	thb	0.709	3.94
grik1	0.658	4.03	thrb	0.810	16.63
hdac2	0.769	7.05	try1	0.863	16.05
hdac8	0.670	1.78	tryb1	0.923	40.08
hivint	0.516	3.05	tysy	0.787	21.44
hivpr	0.697	2.43	urok	0.827	13.04
hivrt	0.529	1.49	vgfr2	0.696	6.38
hmdh	0.738	14.81	wee1	0.909	54.89
hs90a	0.279	0.00	xiap	0.903	29.54

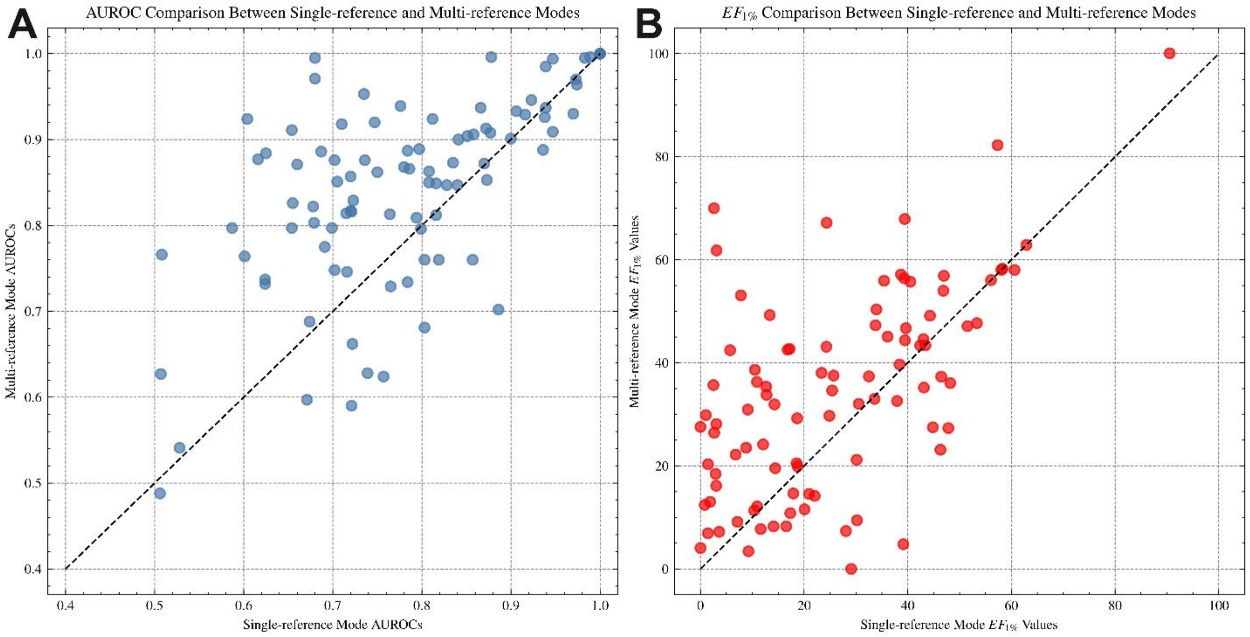

Figure S1. Comparative Analysis of Performance Metrics for AutoDock-SS Single-reference and Multi-reference Modes. A) Illustration of the AUROC values for both modes, with the x-axis representing the AUROC for the Single-reference mode and the y-axis for the Multi-reference mode. Each point represents an individual measurement, and the dashed line indicates the line of equality where the performance of both modes would be identical. B) Illustration of the $EF_{1\%}$ value for the two modes, with the Single-reference mode on the x-axis and the Multi-reference mode on the y-axis. Similar to Panel A, each point signifies an individual data point, and the dashed line represents identical performance across both modes. Data points above the dashed line in both panels suggest a performance advantage for the Multi-reference mode over the Single-reference mode.

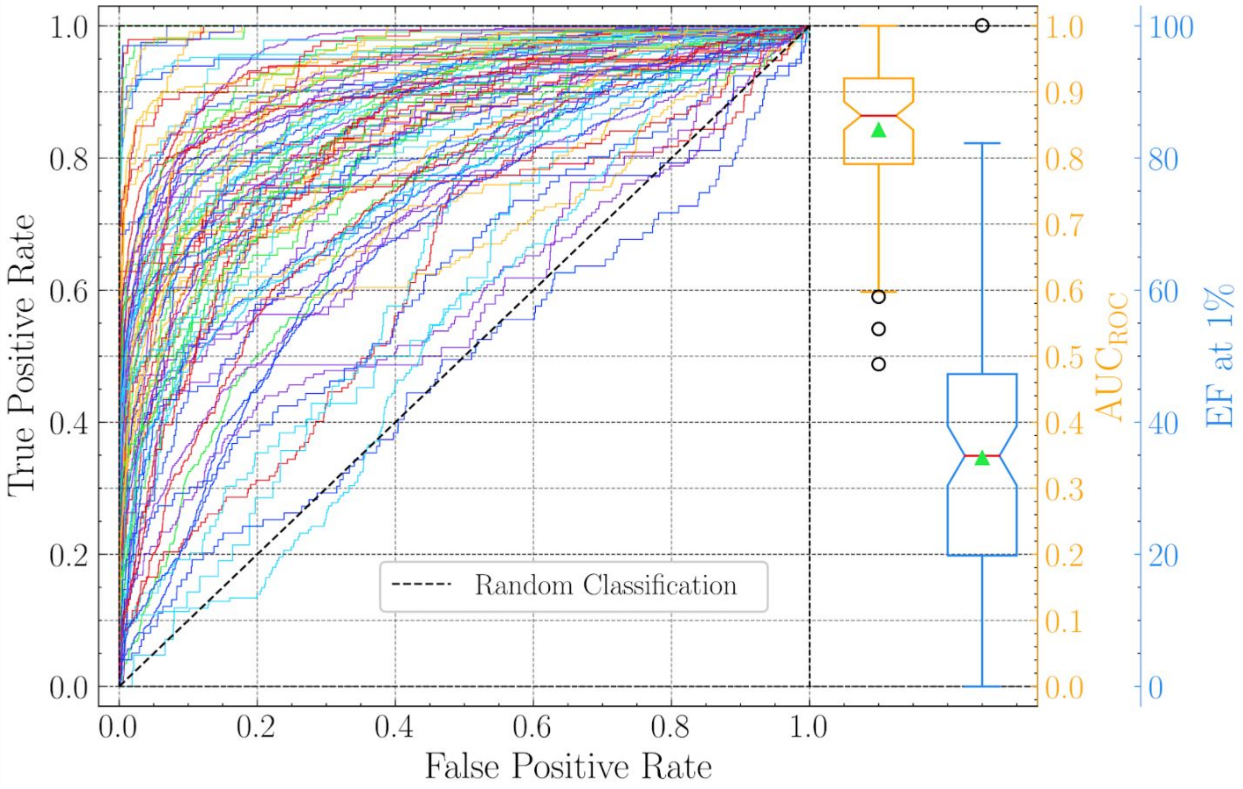

Figure S2. The cluster of 92 ROCs from AutoDock-SS multi-reference mode screening of all DUD-E⁺ targets. The orange boxplot on the right represents the distribution of AUC values, and the blue one shows the distribution of EF1% values. The red line in the boxplot indicates the median AUROC value, and the green triangle represents the mean AUROC value.

Relationship Between Degrees of Freedom and AutoDock-SS Performance: An Analytical Investigation

In our study, we further investigated the impact of the Degree of Freedom (DOF) of library compounds on the performance of AutoDock-SS, utilizing the number of rotatable bonds (NRotB) as a proxy for DOF. Initially, we examined the relationship between the mean and median values of NRotB within the actives and decoys libraries of 102 DUD-E targets and their corresponding AUROC scores. The Pearson correlation coefficients obtained were 0.26 and 0.31 respectively, with p-values equal to 0.02 and 0.00, respectively, indicating a weak positive correlation between both mean and median values of NRotB and AUROC.

Subsequently, a more detailed experiment was conducted. For each target, library compounds were categorized into groups based on the number of NRotB: 0-4, 5-8, 9-12, and over 12. The ratio of actives and decoys in each group was adjusted while maintaining the original ratio of the full actives to decoys libraries using a random selection process. New AUROC values were then recalculated for each group. The compiled results across all DUD-E targets were as follows:

- NRotB 0-4: Mean AUROC of 0.54, Standard Deviation (Std) of 0.15
- NRotB 5-8: Mean AUROC of 0.48, Std of 0.12

- NRotB 9-12: Mean AUROC of 0.48, Std of 0.15
- NRotB Over 12: Mean AUROC of 0.41, Std of 0.23

An Analysis of Variance (ANOVA) test conducted on these results yielded an F-value of 7.82 with a p-value equaled to 0.00. This F-value indicates that there is significant variation among different torsion ranges. It was observed that AutoDock-SS achieved better performance in groups with a lower number of NRotB, and a slight decrease in performance was noted with increasing NRotB.

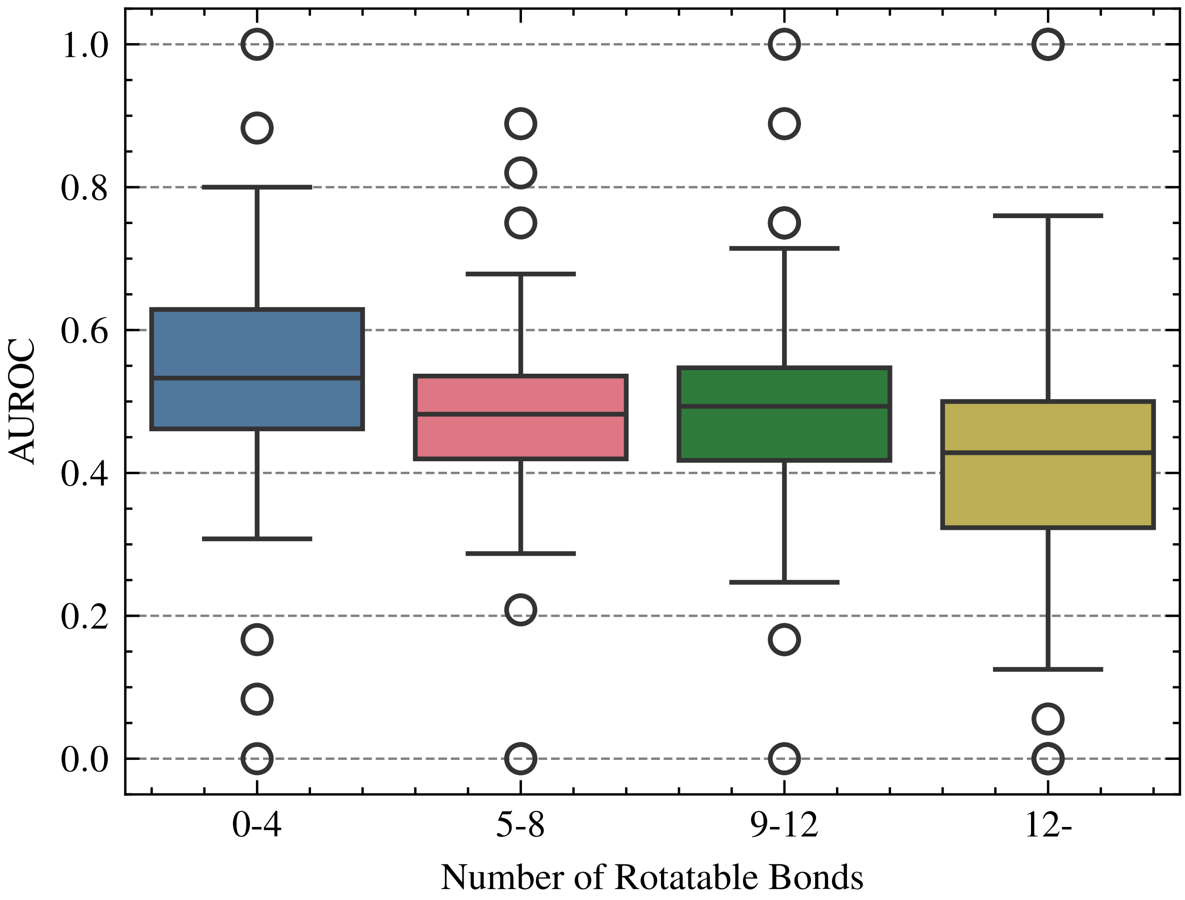

Figure S3. Distribution of AUROC values across different ranges of rotatable bonds. This boxplot displays the variability and central tendency of AUROC values for compounds categorized by the number of rotatable bonds. Outliers indicate compounds with AUROC scores that are notably distinct from the typical score range in each category.
